# Comparison of 24-h Urinary Aldosterone Level and Random Urinary Aldosterone-to-Creatinine Ratio in the Diagnosis of Primary Aldosteronism

**DOI:** 10.1371/journal.pone.0067417

**Published:** 2013-06-28

**Authors:** Che-Hsiung Wu, Ya-Wen Yang, Ya-Hui Hu, Yao-Chou Tsai, Ko-Lin Kuo, Yen-Hung Lin, Szu-Chun Hung, Vin-Cent Wu, Kwan-Dun Wu, the Taiwan Primary Aldosteronism Investigation (TAIPAI) Study Group

**Affiliations:** 1 Division of Nephrology, Buddhist Tzu Chi General Hospital, Taipei Branch, Taiwan; 2 Division of General Surgery, Department of Surgery, National Taiwan University Hospital, Taipei, Taiwan; 3 Division of Endocrine and Metabolism, Buddhist Tzu Chi General Hospital, Taipei Branch, Taiwan; 4 Division of Urology, Buddhist Tzu Chi General Hospital, Taipei Branch, Taipei, Taiwan; 5 Department of Internal Medicine, National Taiwan University Hospital and National Taiwan University College of Medicine, Taipei, Taiwan; Queensland Institute of Medical Research, Australia

## Abstract

**Background:**

Historically, urinary aldosterone level measurement was a commonly employed confirmatory test to detect primary aldosteronism (PA). However, 24-h urine collection is inconvenient and cumbersome. We hypothesized that random urinary aldosterone measurements with correction for creatinine concentration might be comparable to 24-h urinary aldosterone levels (Uald-24 h) in the diagnosis of PA.

**Methods:**

The non-concurrent prospective study was conducted between June 2006 and March 2008 in patients admitted for confirmation of aldosteronism by salt loading test. A 24-h urine sample, which was collected during hospitalization on the day before saline infusion testing after restoration of serum hypokalemia, was collected from all subjects. Moreover, participants were asked to collect a first bladder voiding random urine sample during clinic visits. Uald-24 h and the random urinary aldosterone-to-creatinine ratio (UACR) were calculated accordingly.

**Results:**

A total of 102 PA patients (71 patients diagnosed of aldosterone-producing adenoma, 31 with idiopathic hyperaldosteronism) and 65 patients with EH were enrolled. The receiver operating characteristic curve showed comparable areas under the curves of UACR and Uald-24 h. The Bland-Altman plot showed mean bias but no obvious heteroscedasticity between the two tests. When using random UACR >3.0 ng/mg creatinine as the cutoff value, we obtained a specificity of 90.6% to confirm PA from essential hypertension.

**Conclusions:**

Our study reinforce that the diagnostic accuracy of random UACR was comparable to that of Uald-24 h in PA patients. With the quickness and simplicity of the UACR method and its equivalence to Uald-24 h, this assay could be a good alternative diagnostic tool for PA confirmation.

## Introduction

Primary aldosteronism (PA), characterized by autonomous production of aldosterone, is a common potentially curable disease of secondary hypertension [1]. It affects 5−13% of patients with hypertension in specialized centres [2], [3]. Moreover, PA patients have a high incidence of cardiovascular events in comparison with essential hypertension (EH) patients [4]. As the recognition of PA has increased since the plasma aldosterone-to-renin ratio (ARR) has been used as a screening test, difficulties in establishing a PA diagnosis may be encountered due to atypical manifestations [5]. Caution is necessary regarding the use of ARR because it is largely driven by the plasma renin value. A considerable overlap of the results between patients with PA and those with low-renin EH may be expected, as a high ratio can be due to a low plasma renin activity (PRA) despite a normal plasma aldosterone concentration (PAC) [6]. Physiologically, aldosterone secretion occurs in a pulsatile manner throughout the day, not monotonously the same [7]. Measuring aldosterone in a 24-h urine sample is associated with the advantage of being independent of circadian variations in plasma levels [6], [8]. Therefore, for the purpose of detecting PA, a single 24-h urinary aldosterone level (Uald-24 h) measurement should be a better indicator in clinical practice than a single plasma aldosterone measurement [7], [9]. Historically, the urinary aldosterone level was a commonly employed test for PA diagnosis [10]. The variation in aldosterone excretion throughout the day may negate the use of concentration measurements in random urine collections. However, 24-h urine collections are time consuming and cumbersome [11]. We hypothesized that when properly interpreted by accounting for the effect of different rates of creatinine excretion, the random urinary aldosterone-to-creatinine ratio (UACR) might enable the diagnosis of PA in concordance with the Uald-24 h. The aims of the present study were to investigate the diagnostic accuracy of the UACR compared with Uald-24 h in PA and to define a reliable PA urine test.

## Materials and Methods

### Ethics Statement

The study complied with the Declaration of Helsinki and was approved by the institutional review board of National Taiwan University Hospital (Taipei, Taiwan). All participants signed the informed consent form before inclusion in the study.

### Patient Selection

**Figure 1:**
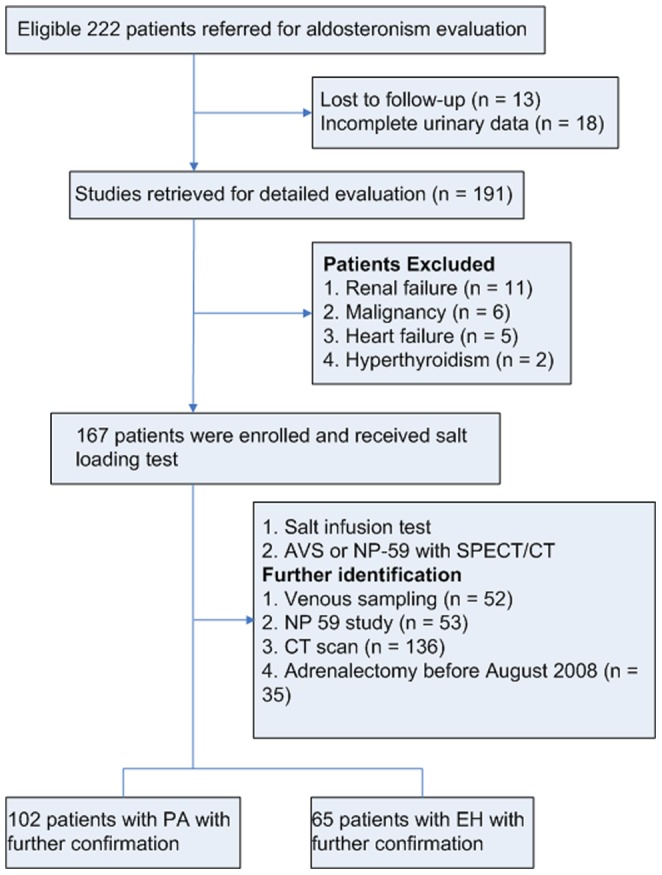
Trial profile. *Abbreviations: AVS, Adrenal venous sampling; CT, computed tomography; EH, essential hypertension; PA, primary aldosteronism; SPE, single-photon emission.

The present study was based on the Taiwan Primary Aldosteronism Investigation (TAIPAI) database. From June 2006 to March 2008, the random urine and 24-h urine samples of the referred patients were routinely analyzed. A total of 222 hypertensive patients were referred to our hypertension clinic for the confirmation of PA after an initial evaluation. The database was constructed for quality assurance in 1 medical center (National Taiwan University Hospital, Taipei, Taiwan) and its 3 affiliated hospitals in different cities (National Taiwan University Hospital Yun-Lin branch, Yun-Lin, southern Taiwan; Tzi-Chi Hospital, Taipei; and Tao-Yuan General Hospital, Tao-Yuan, central Taiwan). All patients hospitalized with the intention to confirm PA diagnosis and who received salt loading test were recruited. Patients were excluded because of loss to follow-up (n = 13), incomplete urinary collection (n = 18), chronic kidney disease with a decreased estimated glomerular filtration rate ([GFR] <60 mL/[min·1.73 m^2^]; n = 11), heart failure, New York Heart Association (NYHA) class II or higher (n = 5), hyperthyroidism (n = 2), and malignancy with metastasis (n = 6) (Fig. 1). All antihypertensive medications were discontinued for at least 21 days before the study. Diltiazem and/or doxazosin were administered for control of marked high blood pressure when required [1]. Medications that might interfere with the renin-aldosterone axis, including steroids, sex hormones, licorice, or non-steroidal anti-inflammatory drugs, were also withheld.

### PA Confirmation and Subtype Studies

All patients with elevated ARR were studied in our unit as inpatients according to our protocol after correcting of serum potassium [12]. After salt loading, a PAC >10 ng/dL was considered a positive diagnosis of PA [13].

A computed tomography (CT) scan of the adrenal glands with a non-ionic iodinated contrast agent was performed in all PA patients, with at least 3-mm contiguous slices obtained in the presence of a normal surround. Dexamethasone suppression adrenocortical scintigraphy (using [I-131] 6-beta-iodomethylnorcholesterol [NP-59]) was performed in patients without a definite adenoma and in those whom bilateral lesions of the adrenal glands were suspected on adrenal CT scans [14].

Adrenal venous sampling (AVS) was performed in patients with equivocal imaging studies or who had a negative imaging study result and a positive salt loading result [15]. Successful venous cannulation was noted if the ratio of the cortisol level of the adrenal vein to that of the inferior vena cava was greater than 3. Lateralization of aldosterone secretion was defined as a >4-fold difference in the aldosterone-cortisol ratio between the bilateral adrenal glands [13].

All operated adenomas were blindly reevaluated by the same pathologist. Histological diagnosis of aldosterone-producing adenomas (APA) was based on the presence of well-defined, encapsulated tumors, predominately consisting of foamy clear cells [16]. Adenomas appear as nodules of clear cells in sheets or nests that are sharply demarcated by a pseudocapsule and compress the non-neoplastic uninvolved adrenal gland. Adenomas are differentiated from nodular adrenal hyperplasia by their solitary and well-circumscribed nature [17], [18]. Hyperplastic adrenal glands are marked by diffuse hyperplasia of cells resembling those of normal zona glomerulosa with or without macronodules or micronodules.

The diagnosis of APA was established in hypertensive patients with elevated ARR, TAIPAI score more than 60% and evidence for lateralized disease by adrenal CT, NP59 scintigraphy or AVS. Idiopathic hyperaldosteronism (IHA) was classified in patients without evidence for lateralized disease as previously report. [12], [19], [20], [21] (Text S1 and figure S1).

### Urinary Studies

A 24-h urine sample (the first urine of the day was included in the 24-h urine sample), which was kept refrigerated until analysis, was collected from all subjects. The urine sample was collected during hospitalization on the day before saline infusion testing after restoration of serum hypokalemia. The daily urine amount was recorded and Uald-24 h was calculated by multiplying the urine aldosterone value by the daily urine amount. When interpreting the results of a 24-h urine collection, we assessed the adequacy of collection by quantifying the 24-h urine creatinine excretion value. The 24-h urine creatinine excretion value was between 15 and 20 mg/kg of body weight.

Moreover, participants were asked to collect a first bladder voiding random urine sample during clinic visits, which primarily were scheduled between 8∶00 and 12∶00 a.m. Antihypertensive medications were still discontinued at the time of first outpatient visit after index discharge, it was within one week after discharge. Diltiazem and/or doxazosin were administered for control of marked high blood pressure when required. The UACR was calculated by dividing the urine aldosterone value (ng/dL) by the simultaneous urine creatinine level (mg/dL).

### Laboratory Measurements

The aldosterone concentration was measured by radioimmunoassays with commercial kits (Aldosterone Maia Kit, Adaltis Italia S.P.A., Bologna, Italy) [1], [20], [22]. The lowest detectable concentration of Aldosterone is 10.0 pg/mL. The normal range of aldosterone is 70–350 pg/mL in the upright position. The PRA was measured by the generation of angiotensin I *in vitro* using a commercially available radioimmunoassay kit (DiaSorin, Stillwater, MN, USA). Its normal range was 2.63±1.32 ng/(mL·h) in the upright position. The mean (standard deviation [SD]) intra- and interassay coefficients of variation (CVs) for the PRA assay were 1.9 (5.0%) and 4.5 (5.2%), respectively.

The urine samples were stored in plastic containers at 4°C. The samples were acid hydrolyzed and then were treated as serum samples during the assay procedure. The sum of free aldosterone and aldosterone from the hydrolysis of aldosterone 18-glucuronide at pH 1 was measured. Tetrahydroaldosterone was not measured in this study.

### Statistical analysis

Statistical analyses were performed with the Statistical Package for the Social Sciences (SPSS) for Windows, version 15.0 (SPSS Inc., Chicago, IL, USA) and MedCalc version 9.0 (MedCalc Software, Mariaherke, Belgium). All data were expressed as the mean ± standard deviation (SD). A normal distribution was attained by appropriate transformations of skewed variables such as aldosterone and ARR. The Bland-Altman plot was used to calculate the differences between the Uald-24 h and UACR against their averages to identify any systemic bias [23]. Since Bland-Altman plot are scale sensitive, both Uald-24 h and UACR was logarithmic transformed to put on the same scale. A Bland -Altman plot would then be used to compare Log (24h-urine aldosterone) and Log (UACR). To test the relationship, the Uald-24 h and UACR were compared using Passing-Bablok regression [24]. The receiver operating characteristic (ROC) curves of the Uald-24 h and UACR were plotted to differentiate patients with PA from those with EH. The optimal cutoff point that led simultaneously to high sensitivity and specificity, and that with the best sensitivity for a specificity >90% were chosen. A p-value of <0.05 was considered significant.

## Results

### Patient Characteristics

A total of 167 hypertensive patients completed the salt loading test and Uald-24 h evaluation. 102 patients had a diagnosis of PA, 71 had APA, and 31 had IHA. The clinical and demographic data of the patients are shown in Table 1. There were no differences in gender, systolic blood pressure, diastolic blood pressure, and body mass index (BMI) between the PA and EH patients. The PA patients were older, had significantly lower serum potassium and PRA levels, and higher aldosterone, ARR, Uald-24 h, and UACR values than those with EH.

**Table 1:** Demographic and clinical data of patients with primary aldosteronism and essential hypertension.

	EH	PA	p-values(EH vs. PA)	APA	IHA	p-values(EH vs. APA)	p-values(EH vs. IHA)
Patients	65	102		71	31		
Men (%)	38 (58.5)	44 (43.1)	0.414	26 (36.6)	18 (58.1)	0.429	0.732
Age(years)	43.1±14.4	47.9±11.1	0.04	47.2±11.8	49.6±9.2	0.184	0.014
BMI (kg/m^2^)	24.6±4.2	25.3±3.4	0.186	25.2±3.7	25.6±2.7	0.563	0.041
Potassium (mmol/L)	4.3±0.5	3.6±0.8	0.001	3.5±0.8	3.8±0.7	<0.001	0.164
Creatinine (mg/dL)	0.98±0.17	0.98±0.62	0.064	1.01±0.73	0.89±0.21	0.028	0.286
PRA (ng/[mL·hr])	3.84±7.57	0.93±2.69	0.004	1.10±3.17	0.53±0.76	0.009	0.001
Aldosterone (ng/dL)	38.0±35.4	49.3±31.4	0.033	47.5±28.0	53.5±38.3	0.085	0.055
ARR	136.6±457.0	1000.9±2120.0	<0.001	975.8±2303.8	1057.7±1664.3	0.004	0.005
sBP (mmHg)	144.4±18.5	150.9±20.1	0.551	152.1±21.5	148.2±16.7	0.362	0.887
dBP (mmHg)	86.0±12.5	93.4±15.6	0.213	94.1±16.4	91.7±14.6	0.104	0.963
Uald-24 h (ug/day)	11.6±6.5	20.1±8.7	0.001	20.5±8.3	19.2±9.6	0.012	<0.001
UACR	1.33±1.10	3.17±2.32	<0.001	3.49±2.08	2.42±2.69	<0.001	<0.001

* Abbreviations: APA, aldosterone-producing adenoma; ARR, aldosterone-renin ratio; BMI, body mass index; dBP, diastolic blood pressure; EH, essential hypertension; IHA, idiopathic hyperaldosteronism; PRA, plasma renin activity; sBP, systolic blood pressure; UACR, random urinary aldosterone-to-creatinine ratio; Uald-24 h, 24-h urinary aldosterone level.

* Data are expressed as mean ± standard deviation (SD) unless otherwise indicated.

### The Relationship between UACR and Uald-24 h

**Figure 2:**
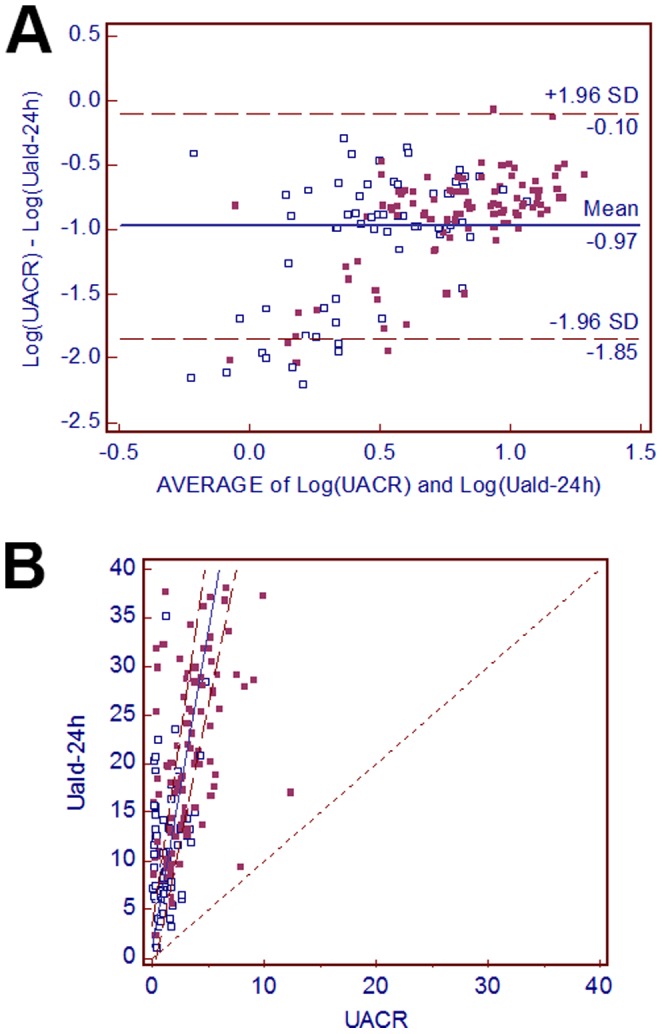
Comparison of random urinary aldosterone-to-creatinine ratio (UACR) and the 24-h urinary aldosterone level (Uald-24 h). (a) Bland-Altman plots to compare Log (UACR) and Log (Uald-24 h) and (b) Passing-Bablok regression scatter plot. Solid line, regression line; dashed lines, 95% confidence interval (CI) for the regression line; dotted line, line of identity.

The Bland-Altman plot (Fig. 2a) showed mean bias but no obvious heteroscedasticity between the Uald-24 h and UACR. Passing-Bablok regression analysis (Fig. 2b) revealed an intercept of 0.8547 [95% CI, −1.4964 to 2.9204] and slope of 6.6214 (95% CI, 5.5863−8.0080), suggesting no constant distance (interval of Uald-24 h and UACR, including 0) but a proportional difference (slope >1) between the 2 methods.

### The Performance of UACR and Uald-24 h Assessment in Patients with PA and EH

**Figure 3:**
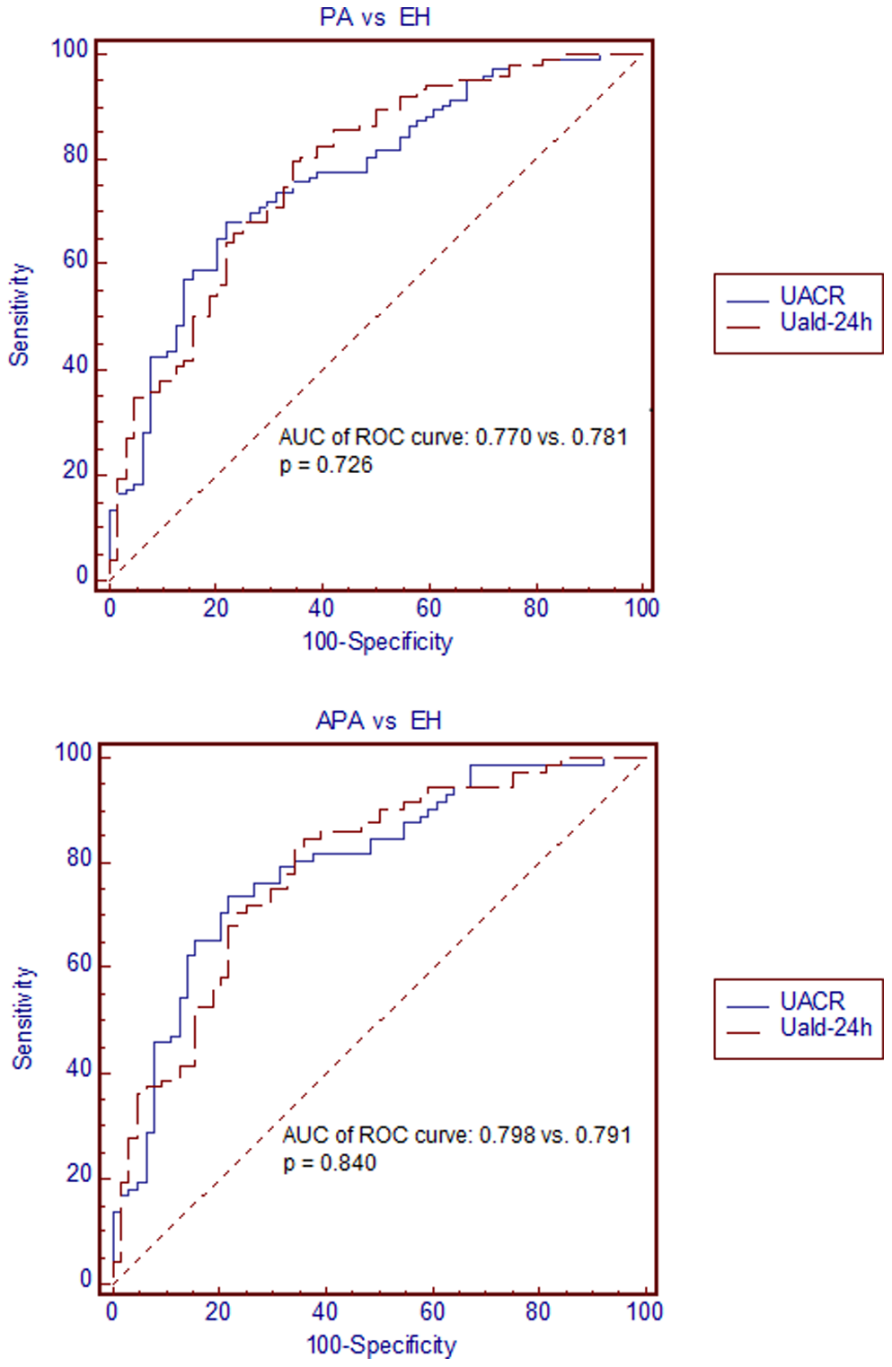
Receiver operating characteristic (ROC) curves for the diagnosis of primary aldosteronism by UACR and Uald-24 h. Abbreviations: APA, aldosterone-producing adenoma; AUC, area under the curve; EH, essential hypertension; PA, primary aldosteronism; UACR, urinary aldosterone-to-creatinine ratio; Uald-24 h, 24-h urinary aldosterone level.

The ROC curve, illustrating the sensitivity and specificity of the UACR and Uald-24 h for detecting suspicious PA in patients with hypertension, was plotted to identify the optimal cut-off value for differential diagnosis between EH and PA (Fig. 3). The optimal cut-off values of the UACR were 2.62 ng/mg and that of the Uald-24 h was 16.4 µg. The thresholds with the best sensitivity for a specificity of 90.6% of the UACR were 3.0 ng/mg and that of the Uald-24 h was 20.3 µg. The calculations and accuracy measures of the screening tests, including the proposed UACR and Uald-24 h assessments, are given in Table 2. The areas under the curve were 0.77±0.036 and 0.781±0.035 for the UACR and Uald-24 h, respectively, without a significant difference (p = 0.726; Table 2 and Fig. 3). Likewise, the ROC curve showed that the UACR and Uald-24 h provided a comparable ability to differentiate PA from EH.

**Table 2:** Diagnostic performance in patients with EH and PA.

Operative features	PA vs. EH		APA vs. EH	
	UACR	Uald-24 h	UACR	Uald-24 h
AUC of ROC curve	0.77	0.781	0.798	0.791
Optimal cutoff value	2.62	16.4	2.2	15.8
Positive predictive value (%)	88.7	84.2	82.2	80.3
Negative predictive value (%)	54.3	57.1	71.6	69.3
Positive likelihood ratio	4.88	3.31	4.12	3.63
Negative likelihood ratio	0.52	0.47	0.35	0.39
Sensitivity (%)	53.4	62.1	70.8	68.1
Specificity (%)	89.1	81.2	82.8	81.2
Cutoff value with specificity >90%	3.01 (Sen 44.7 Spe 90.6)	20.3 (Sen 41.8 Spe 90.6)	3.01 (Sen 51.4 Spe 90.6)	20.3 (Sen 44.4 Spe 90.6)
Accuracy (%)	67.1	69.4	76.5	74.3

* Abbreviations: APA, aldosterone-producing adenoma; AUC, area under the curve; EH, essential hypertension; PA, primary aldosteronism; ROC, receiver operating characteristic curve; Sen, sensitivity; Spe, specificity; UACR, urinary aldosterone-to-creatinine ratio; Uald-24 h, 24-h urinary aldosterone level.

In a subgroup analysis of APA vs. EH, the optimal cut-off value of the UACR was 2.2 ng/mg, and that of the Uald-24 h was 15.8 µg. The thresholds with the best sensitivity for a specificity of 90.6% of the UACR were 3.0 ng/mg and that of the Uald-24 h was 20.3 µg (Table 2). Consistent with the previous finding, the areas under the curve for the UACR and Uald-24 h did not differ significantly (UACR, 0.798±0.038 vs. Uald-24 h, 0.791±0.038; p = 0.84).

## Discussion

Our results reinforce the view that measurement of the UACR can be a substitute for the Uald-24 h in the diagnosis of PA, as it is easier to perform and provides results that are comparable to those of the Uald-24 h.

In a recently published Aliskiren in the eValuation of prOteinuria In Diabetes (AVOID) substudy, the Uald-24 h was chosen over PAC to assess the effects of direct renin inhibition with aliskiren in combination with losartan, as the urinary analysis better reflects the mean aldosterone level than random plasma samples due to the diurnal variation of plasma aldosterone levels [25]. Nevertheless, this urine collection method is inconvenient and cumbersome, and it is often difficult to collect a complete 24-h urine sample accurately. Our study demonstrated that the UACR is a comparable alternative to the Uald-24 h for the diagnosis of PA, particularly at lower Uald-24 h levels. Once-only urine collection is an easy and reliable method that can be performed in an outpatient setting.

The elevation of the ARR is predominantly an indicator of low PRA if PRA result is used as measured instead of setting a minimal value [3], [26], and its specificity is relatively low [27] and further confirmatory testing is always necessary to establish the diagnosis of PA [28]. In addition, due to fluctuations in the PAC and PRA, a single ARR result within the reference range does not exclude hyperaldosteronism and repeated measurements may be required in the clinical setting. The solution could lie in the determination of aldosterone excretion from urine samples, which has proven useful previously. Historically, the Uald-24 h, instead of PAC, is used as a confirmatory test in the diagnosis of PA [29]. Less than 5% of secreted aldosterone is excreted as free aldosterone into the urine, whereas approximately 10% is excreted as aldosterone-18-glucuronide produced primarily in the kidney [30]. Tetrahydroaldosterone, produced after metabolization in the liver, accounts for a higher proportion of aldosterone excretion, but nonetheless accounts for only one-third of total aldosterone excretion. Some aldosterone metabolites are unknown or are not determined in clinical practice [31]. This likely explains why the results of some studies showed that urinary aldosterone determination does not have a better diagnostic value than plasma determinations. Schirpenbach et al. [8] reported that the mean urinary excretion levels of tetrahydroaldosterone and aldosterone-18-glucuronide were significantly higher in PA patients compared to EH patients and normal subjects. However, substantial overlaps were observed between patients with PA and the 2 other groups. While employing appropriate cutoff values, urinary metabolites were demonstrated to have a high specificity for primary aldosteronism, but owing to a high level of overlap in the lower range of values, a rather low sensitivity was observed. Likewise, our study showed a similar result, as urinary aldosterone measurement had a high specificity, but a low sensitivity in diagnosing PA. In fact, urinary aldosterone assessment was traditionally employed as a confirmatory test in the diagnosis of PA [10]. As a confirmatory test, urinary aldosterone concentration measurement would provide a high specificity and positive predictive value, rather than a high sensitivity, which is essential for a screening test (such as ARR); such characteristics were consistent with both the Uald-24 h and UACR evaluations according to our data. We found that the best cutoff value with the highest sensitivity at specificity more than 90% for identifying patients with PA was 20.3 µg/24 h for the Uald-24 h. This result was reasonable, as the normal values for urinary aldosterone range from 3 to 15 µg/24 h in the literature [29]. Martinerie and colleagues found that in term neonates, random urinary aldosterone levels (corrected for creatinine excretion), unlike plasma aldosterone levels, were significantly and negatively correlated with the plasma potassium levels. They suggested that urinary aldosterone measurement (pg/µg creatinine) is the best index for accurate evaluation of mineralocorticoid effector mechanisms [32]. Moreover, Djajadiningrat et al. found that using the UACR for an oral fludrocortisone suppression test may be useful for the diagnosis of PA in cats [33]. To the best of our knowledge, this is the first study of whether the random UACR assessment can be used as a diagnostic tool for PA in humans. Our results, evaluated using the ROC curves, showed that the UACR was a reliable alternative to the Uald-24 h. We proposed a cutoff value to use the UACR to diagnose PA, which had not been previously available. In effect, these cutoff values offered acceptable specificity as required by a confirmatory test after an initial screening test. In addition, since the intended clinical use of urinary aldosterone is to confirm PA, thresholds with a high specificity are needed. We also provided thresholds to use the UACR and Uald-24 h to diagnose PA with specificity more than 90% with related sensitivity. Nevertheless, further study to compare the UACR against other suppression tests in patients with clinical clues for PA is necessary.

### Conclusions

In summary, UACR measurement can be a substitute for Uald-24 h in the diagnosis of PA because it is easier to perform and provides comparable results to those of Uald-24 h assessment. We believe that urinary aldosterone determination will play an important role in the diagnosis of PA. This simple urine screening method could be performed on an outpatient basis with easy performance, rapid assessment, and lower health care costs. UACR measurement should be further verified and integrated to refine the diagnostic workup for PA.

## Supporting Information

Figure S1 **The differential subtyping protocol of the TAIPAI group.** *Abbreviations: AVS, adrenal venous sampling; APA, aldosterone-producing adenomas; IHA, idiopathic hyperaldosteronism; NP-59 SPECT/CT, I131-6b-iodomethyl-19-norcholesterol/SPECT/CT; INC, incidentaloma.
(TIF)

Table S1 **Diagnostic performance of ARR and UACR in patients with EH and PA.**
(DOC)

Text S1 **The differential subtyping protocol of the TAIPAI group.**
(DOC)
