# Supplementary figures and images for: Comparison of 24-h Urinary Aldosterone Level and Random Urinary Aldosterone-to-Creatinine Ratio in the Diagnosis of Primary Aldosteronism
Source: PLoS One. 2013 Jun 28;8(6):e67417. doi: 10.1371/journal.pone.0067417 (PMC3696056; doi:10.1371/journal.pone.0067417)

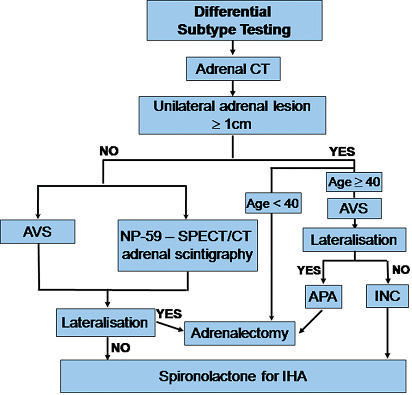

Supplement: Figure S1 — The differential subtyping protocol of the TAIPAI group. *Abbreviations: AVS, adrenal venous sampling; APA, aldosterone-producing adenomas; IHA, idiopathic hyperaldosteronism; NP-59 SPECT/CT, I131-6b-iodomethyl-19-norcholesterol/SPECT/CT; INC, incidentaloma. (TIF) [file pone.0067417.s001.tif]
