# Supplementary material for: Comparison of 24-h Urinary Aldosterone Level and Random Urinary Aldosterone-to-Creatinine Ratio in the Diagnosis of Primary Aldosteronism
Source: PLoS One. 2013 Jun 28;8(6):e67417. doi: 10.1371/journal.pone.0067417 (PMC3696056; doi:10.1371/journal.pone.0067417)
Supplement: Table S1 — Diagnostic performance of ARR and UACR in patients with EH and PA. (DOC) [file pone.0067417.s002.doc]

Table S1. Diagnostic performance of ARR and UACR in patients with EH and PA

| Operative features | | **PA vs. EH** | | **APA vs. EH** | |
| --- | --- | --- | --- | --- | --- |
|  |  | UACR | ARR | UACR | ARR |
| AUC of ROC curve | | 0.77 | 0.786 | 0.798 | 0.767 |
| Optimal cutoff value | | 2.62 | 33.65 | 2.2 | 33.65 |
| Positive likelihood ratio | | 4.88 | 2.27 | 4.12 | 2.26 |
| Negative likelihood ratio | | 0.52 | 0.18 | 0.35 | 0.18 |
| Sensitivity (%) | | 53.4 | 89.3 | 70.8 | 88.9 |
| Specificity (%) | | 89.1 | 60.7 | 82.8 | 60.2 |
