# Supplementary material for: Comparison of 24-h Urinary Aldosterone Level and Random Urinary Aldosterone-to-Creatinine Ratio in the Diagnosis of Primary Aldosteronism
Source: PLoS One. 2013 Jun 28;8(6):e67417. doi: 10.1371/journal.pone.0067417 (PMC3696056; doi:10.1371/journal.pone.0067417)
Supplement: Text S1 — The differential subtyping protocol of the TAIPAI group. (DOC) [file pone.0067417.s003.doc]

**Supplementary**

Material and methods

1. The diagnosis of aldosterone-producing adenomas (APA) was established in hypertensive patients and required the following criteria: . (Fig S1) : (1) evidence of autonomous excess aldosterone production based on an aldosterone-to-renin ratio (ARR) > 35 and TAIPAI score more than 60%; and post-saline loading plasma aldosterone concentration (PAC) > 10 ng/dl, (2) lateralization of aldosterone secretion at adrenal venous sampling (AVS) or during dexamethasone suppression NP-59 SPECT/CT; at pre-operative evaluation., (3) evidence of adenoma at CT scan; and (4) pathologically proven adenoma after an adrenalectomy if operated, and cure of hypertension without anti-hypertensive agents or improved hypertension, potassium, PAC, and plasma renin activity (PRA). The flow diagram of the enrollee has been further listed.
2. Idiopathic hyperaldosteronism (IHA) was classified by the following criteria: (1) evidence of autonomous excess aldosterone production based on an ARR > 35 and TAIPAI score more than 60%; and post-saline loading PAC > 10 ng/dl, (2) non-lateralization of aldosterone secretion at AVS or during dexamethasone suppression NP-59 SPECT/CT; at pre-operative evaluation, (3) evidence of bilateral diffuse enlargement on CT scan; and/or (4) evidence of diffuse cell hyperplasia in the pathology studies if operated.

Reference

1. Rossi GP, Belfiore A, Bernini G, Desideri G, Fabris B, et al. (2007) Comparison of the captopril and the saline infusion test for excluding aldosterone-producing adenoma. Hypertension 50: 424-431.

2. Wu VC, Chang HW, Liu KL, Lin YH, Chueh SC, et al. (2009) Primary Aldosteronism: Diagnostic Accuracy of the Losartan and Captopril Tests. Am J Hypertens 22: 821-827.

3. Kuo CC, Wu VC, Huang KH, Wang SM, Chang CC, et al. (2011) Verification and evaluation of aldosteronism demographics in the Taiwan Primary Aldosteronism Investigation Group (TAIPAI Group). J Renin Angiotensin Aldosterone Syst 12: 348-357.

4. Wu VC, Yang SY, Lin JW, Cheng BW, Kuo CC, et al. (2011) Kidney impairment in primary aldosteronism. Clin Chim Acta 412: 1319-1325.

5. Yen RF, Wu VC, Liu KL, Cheng MF, Wu YW, et al. (2009) 131I-6beta-iodomethyl-19-norcholesterol SPECT/CT for primary aldosteronism patients with inconclusive adrenal venous sampling and CT results. J Nucl Med 50: 1631-1637.

6. Wu VC, Chao CT, Kuo CC, Lin YH, Chueh SC, et al. (2012) Diagnosis and Management of Primary Aldosteronism. Acta Nephrologica 26: 111-120.
